# Investigation of the steady state measurement process

**DOI:** 10.1155/S1463924688000197

**Published:** 1988

**Authors:** J. L. Nagy, L. Leisztner, K. M. Hangos

**Affiliations:** 1 Institute of Forensic Science PO Box 314/4 Budapest H 1903 Hungary; 2 Computer and Automation Institute Hungarian Academy Sciences PO Box 63 Budapest H 1502 Hungary

## Abstract

Based on the role of steady state concept in the model of analytical chemical measurement and deduction, the definition of ‘practically sleady slate’ (PSS) has been inlroduced. The defnition does not require the process to be in steady state in a strictly mathematical sense. In order to fulfil the requiremenls of ‘practically steady state’ the random error and the syslematic error must vary within a suitable limit, and the expected fgure for the measured value must be within a specified range.

The goal of the present investigation was to detect the steady state of the measurement process with respect to the analytical information (peak area ratio) based on the measured values. The method proposed proved to be useful for the determination of the simultaneously present systematic error and random error. Control based on the measured values of the internal standard is useful, but additional information is necessary. There are several advantages to the method described: the results for the internal standard indicate possible sources of disturbances and allow the end of the steady state measurement process to be predicted.


Journal of Automatic Chemistry, Vol. 10, No. 2 (April–June 1988), pp. 101–105

### Investigation of the steady state measurement process

**J. L. Nagy, L. Leisztner**
*Institute of Forensic Science, H 1903 Budapest, PO Box 314/4, Hungary*
**and K. M. Hangos**
*Computer and Automation Institute, Hungarian Academy Sciences, H 1502 Budapest, PO Box 63, Hungary*

*Based on the role of steady state concept in the model of analytical chemical measurement and deduction, the definition of 'practically steady state' (PSS) has been introduced. The definition does not require the process to be in steady state in a strictly mathematical sense. In order to fulfil the requirements of 'practically steady state' the random error and the systematic error must vary within a suitable limit, and the expected figure for the measured value must be within a specified range.*

*The goal of the present investigation was to detect the steady state of the measurement process with respect to the analytical information (peak area ratio) based on the measured values. The method proposed proved to be useful for the determination of the simultaneously present systematic error and random error. Control based on the measured values of the internal standard is useful, but additional information is necessary. There are several advantages to the method described: the results for the internal standard indicate possible sources of disturbances and allow the end of the steady state measurement process to be predicted.*

### Introduction

It is important to know the reliability (whether or not it is in steady state) of any measurement process (i.e. sample preparation, measurement and data processing). This information is especially important in automated chemical analyses where investigations of the measurement process state have to be carried out automatically.

The majority of the tests for the steady state hypothesis [1 and 2] will detect only deterministic variations and they can only be used in estimate random errors. Moreover, these methods do not allow the simultaneously present systematic error and random error to be investigated separately.

Control of the steady state measurement process can be carried out in various ways, for example with the help of control samples or on the basis of measurement results of the internal standard [3 and 4].

In this paper the role of steady state concept in the information theory model of analytical chemical measurement and deduction is discussed. The definition of the concept of practically steady state is also given.

A method for investigation of analytical chemical measurement processes which is able to separately signal the systematic error and the random error is introduced. In the course of experimental testing of the model, sample sequences consisting of control samples are measured. Based on the measured values of the internal standard, the possibility of detection the steady state measurement process is investigated with respect to the analytical information (chromatography peak area ratios in this case).

### The role of the steady state concept in the information theory model

For the evaluation of the steady state measurement process in chemistry, it is necessary to have a mathematical model which fits the general information theory model of analytical chemical measurement and deduction. According to the known information theory model of analytical chemical measurement, analytical information is produced in most cases by the measurement of samples and by primary data processing [5]. If the composition of the sample is known, it can provide analytical knowledge from the analytical information. This is called calibration. In other cases the composition of the unknown sample (chemical information) can be deduced from the measured analytical information with the help of analytical knowledge.

The measurement process can be considered to be in steady state if the process of the production of chemical information is steady state. For the sake of simplicity this can be kept under control by the investigation of the process of analytical information production.

The measurement error can be interpreted in the space of analytical information (figure 1) provided by measuring the chemical information is denoted by $\mathcal{M}(\underline{K})$, where $\mathcal{M}$ is the transformation which is characteristic of the measurement system and the evaluation method. In this case the measurement error in the analytical information is:

$$\underline{h}_a = \underline{a} - \mathcal{M}(\underline{K})$$

where $\underline{a}$ is the measured value of the analytical information. In the following the model produced from analytical information will be used, because $\underline{h}_a$ can be easily computed. Furthermore $\underline{h}_a$ involves only the error of the analytical measurement (and evaluation) and does not take into account the error of the analytical deduction. The measurement error interpreted in the space of chemical information ($\underline{h}_k$) is

$$\underline{h}_K = \underline{K}^* - \underline{K} = [\tilde{\mathcal{M}}^{-1}\{(\underline{K}) + \underline{h}_a\} + \underline{h}_K^*] - \underline{K}$$

It can be seen that $\underline{h}_K$ depends, in a complex way, both on the error of measurement ($\underline{h}_a$) and deduction ($\underline{h}_K^*$). $\tilde{\mathcal{M}}^{-1}$ is generally a non-linear transformation, which is characteristic of inverse operation of the calibration. This can be derived only in approximate terms.

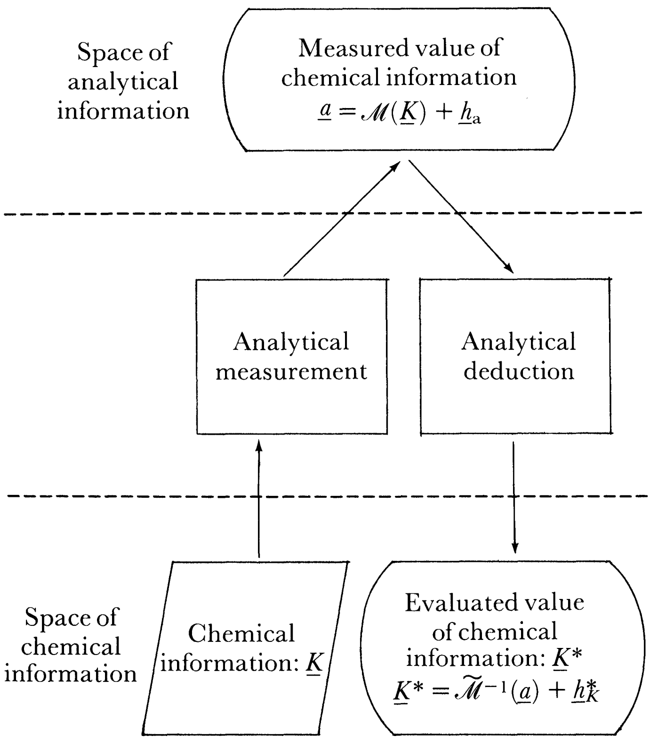

$\underline{h}_a$ : the measurement error of the analytical information.
$\underline{h}^*_K$ : the error of the analytical deduction.
$\mathcal{M}(.)$: the transformation characterizing the measurement and the evaluation.

*Figure 1. The information theory model of the analytical chemical measurement and deduction based on calibration.*

### The mathematical model of the steady state measurement process

In order to decide if the measurement process can be considered to be in steady state or not, measurement error processes of continuous and discrete time samples must be investigated.

It is assumed that the process under study can be characterized by a single variable $x(t)$. We suppose that this variable varies according to a continuous time stochastic process, with continuous trajectories with probability 1.

$x(t)$ can be described by the sampled form of the Ito-process [6]:

$$x_k - x_{k-1} = v_k + e_k \quad k = 1, 2 \ldots \tag{1}$$

where an equidistant-delta function is applied in order to sample with a

$$T = \tau_k - \tau_{k-1}$$

sampling interval.

In equation (1) $x(k)$ is the measured value of the sampled sequence. $v_k = m \cdot T$ is the deterministic error term and $e_k$ is a normally distributed white noise sequence with zero mean and $s \cdot T$ variance:

$$e_k \sim N(0, s \cdot T)$$

($m$ and $s$ are constants).

The measurement process described by the stochastic sequence $\{x_k\}_{k=1}^N$ is called steady state if the elements of $x_k$, as random variables, have the same distribution for every $k$. The above notion of steady state is an abstraction which will never exist in reality. This ideal state can be only approximated during the measurement. Therefore, on investigating the measurement process, an answer can only be found if the given conditions are adequately approximated.

The stochastic sequence described by equation (1) can be regarded to be in practically steady state (PSS) if the parameters $v_k$, $e_k$ and $x_k$ fulfil the following inequalities:

$$\begin{gathered} |m| \leq m^* \\ s \leq s^* \\ z_1 \leq E[x_k] \leq z_2 \end{gathered} \tag{2}$$

where $m^*$, $s^*$, $z_1$ and $z_2$ are constants.

The measurement process has been characterized by the stochastic sequence $\{x_k\}_{k=1}^N$, and for this reason the measurement process will be considered to be in PSS if the sequence $\{x_k\}_{k=1}^N$ is in PSS too.

The definition (2) does not require the process to be in steady state in a strict mathematical sense; rather, it requires only that the parameters $m$ and $r$ be 'small enough' in absolute value. This means that both systematic and random error can change, but only within given limits.

The first two inequalities of the definition (2) agree with inequalities described by Almásy and Hangos [7]. The third inequality is reasonable because the deterministic error can change in the course of PSS.

This means that the third inequality leads to the expected value of the measured value ($E[x_k]$) being in a given range.

To detect whether the systematic error or random error has changed within the acceptable limit, the Student test and the $\kappa^2$ test have been applied. The independence of the consecutive measurement results was controlled by computing the autocorrelation function.

In the case of slowly varying processes the technique of exponential forgetting [8] can be applied. This means that the new statistics of the process are computed from the old ones and from the new data in such a way that the old statistics are multiplied by a weighting factor which is less than 1. Therefore the old data are conserned by an exponentially decreasing weighting factor.

The application of exponential forgetting enables the fulfilment of conditions of definition (2) to be investigated in $n$ samples. The forgetting factor ($Q$) determines the number of statistically equivalent samples ($n = 1/(1 - Q)$)

forming a moving window. The parameters $m$ and $s$ are considered to be constants within these $n$ samples. The changes in these parameters in comparison with the previous stage are investigated within given limits. The constants $Q$, $m^*$, $s^*$, $z_1$ and $z_2$ have to be determined experimentally.

### Testing the model

The measurement system was used in a blood ethyl alcohol determination. The sample sequences containing control samples were analysed with a Fractovap 4200 (Carlo Erba, Milan, Italy), which was equipped with an HS 250 automatic head space sampler (Carlo Erba). The chromatographic data was collected and evaluated with an HP 3354 B/C Laboratory Automation System (Hewlett-Packard, California, USA).

Measurements were performed under the following chromatographic conditions:

*Column*: 1.5 m long, 0.2 cm in diameter Carbopack C (80/100 mesh) coated with 0.2% Carbowax$^R$ 1500 (Supelco Inc., Bellefonte, Pennsylvania, USA).

*Liquid samples' volume*: 0.4 ml.
*Gas samples' volume*: 0.8 ml.
*Sampling vials' volume*: 5.0 ml.

*Injection syringes*: Hamilton gas-tight syringes 1001 LTSN (1 ml) (Hamilton, Bonaduz, Switzerland).

*Temperatures* were:

| | |
|---|---|
| injection syringe: | 95 °C |
| equilibrium vapor phase: | 50 °C |
| injector: | 125 °C |
| detector: | 125 °C |
| column oven: | 80 °C. |

The *carrier gas* ($N_2$) flow rate was 100 ml/min.
Injection septa: Tightsep 11 mm in diameter and Thermogreen L–B–2 12.5 mm in diameter (Supelco Inc., Bellefonte, Pennsylvania, USA).

The control samples' composition in distilled water:

1 and 2 g/l ethyl alcohol (chrom. E. Merck, Darmstadt, FR Germany)
1,6 g/l 1-propanol (chrom. E. Merck, Darmstadt, FR Germany)
100 g/l sodium sulphate (alt. Reanal, Budapest, Hungary)

A typical chromatogram is shown in figure 2.

The influence of controllable parameters (sample composition change, injection septum and injection syringe ruin) was investigated in order to determine which of these cause the cessation of the steady state. The chromatographic parameters were strictly the same in the course of experiments. To avoid systematic error due to sample preparation stock solutions were applied.

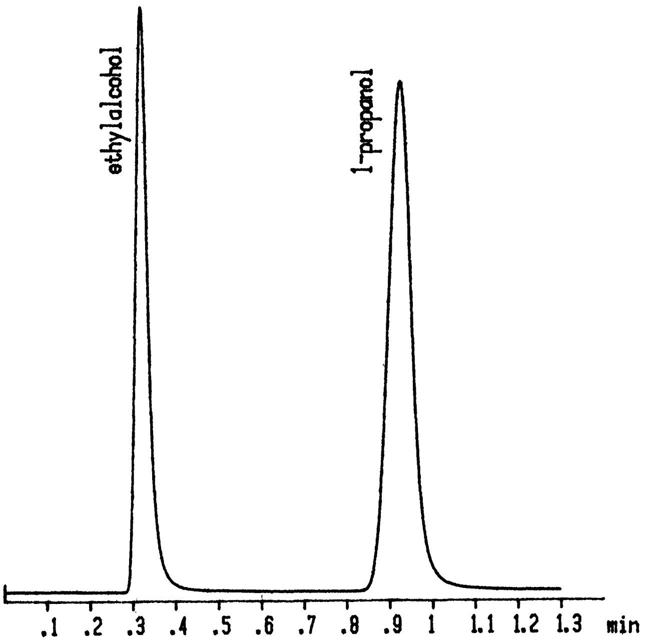

*Figure 2. Typical chromatogram of the ethyl alcohol determination (see text for the chromatographic conditions).*

In order to determine the effect of the small changes of sample composition, the samples were prepared from three stock solutions which had nearly the same composition. In order to decrease the number of samples necessary for leaving the steady state—which would have been more than 10 000—the control samples were measured and evaluated in series of 20 samples. After each series 100 blank injections without a sample were performed. For the sake of clarity the value of $x_k$ was the average of 20 measurements according to the measurement sequences.

The reason for the cessation of steady state was controlled after the end of the measurement series. For this purpose the measurement process was investigated after the change of the injection septum, and then following the change of the injection septum and syringe simultaneously.

In order to model the effect caused by a damaged septum another measurement sequence was performed. This measurement sequence was carried out with Thermogreen L–B–2 septum, which is unsuitable for this task. Therefore in this case the measurement process left the PSS within a relatively short time. In this measurement sequence the value of $x_k$ was the result of each sample.

### Results

The typical results of the measurement sequence with Tightsep septa (which are suitable for the task) are shown in figures 3 and 4. The average of peak area ratios of samples in series of 20 is plotted against the serial number of the sample sequence (figure 3). The influence of sample composition changes are clearly seen. There are two jumps after the 30th and 81st series (about 5% with

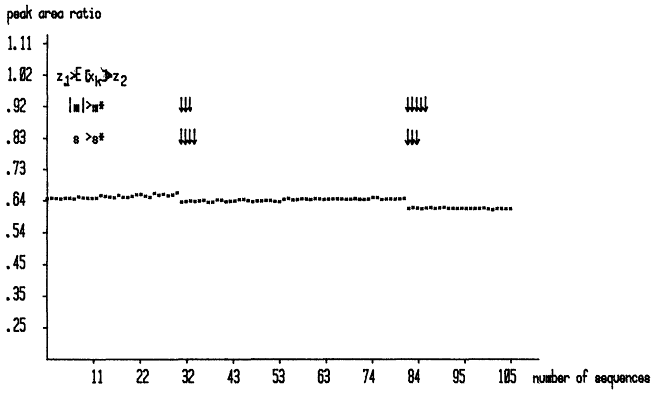

*Figure 3. The results of PSS test investigation to the peak area ratios (suitable septum for the task). Each point represents the average of 20 measurements, the arrows correspond to failure of the steady state.*

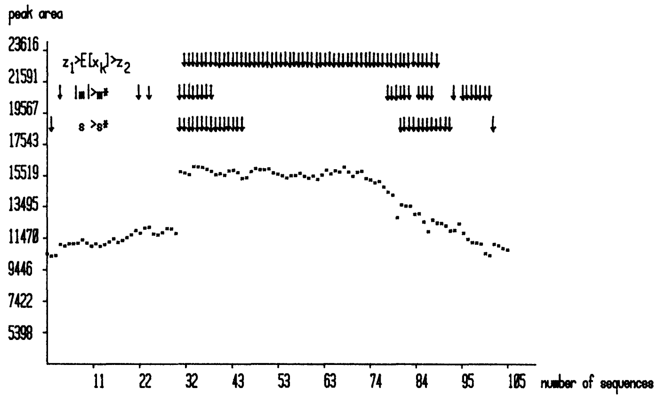

*Figure 4. The results of PSS test investigation to the peak area of internal standard (suitable septum for the task). Each point represents the average of 20 measurements, the arrows correspond to failure of the steady state.*

respect to the measured value) in the results of peak area ratios. Disregarding the two jumps there were no indications that the system failed to meet the criteria of the PSS test for area ratios.

Figure 4 shows the measurement results of the internal standard. The sample composition changes, due to an error in sample preparation, caused jumps (however, the internal standard area does not neccessarily indicate such changes). After the 30th sequence the jump really can be easily observed in the illustration (about 30% with respect to the measured value), but the influence of stock solution change is not shown after the 81st sequence. The reason for this is that the measurement sequence left PSS soon after the 77th sequence; the standard deviations increased and the internal standard areas, and the quantity of the measured samples, respectively, decreased.

This deterioration is due either to the injection septum or syringe, or to both. To determine the reason for the decrease in measured sample the following experiments were performed. Firstly, the measurement process was investigated under the conditions holding after the 105th series (see table 1[*a*]). This was followed by septum change and two more series (table 1[*b*]). Then the injection septum and syringe were both changed together and the two series were measured (table 1[*c*]).

*Table 1. The effects of changes of septum and both septum and syringe on the peak area of the internal standard and the peak area ratio.*

| Sequence | Area of internal standard | Rel. SD% | Peak area ratio | Rel. SD% |
|---|---|---|---|---|
| | (*a*) After 105 sequences | | | |
| 1. | 10756 | 2·24 | 0·613 | 0·22 |
| 2. | 10706 | 2·43 | 0·614 | 0·20 |
| | (*b*) After the change of the injection septum | | | |
| 1. | 9360 | 8·52 | 0·620 | 0·44 |
| 2. | 9182 | 5·36 | 0·615 | 0·40 |
| | (*c*) After the change of the injection septum and syringe. | | | |
| 1. | 15054 | 1·13 | 0·622 | 0·17 |
| 2. | 15478 | 1·24 | 0·619 | 0·10 |

From the data in table 1 it can be seen that the standard deviations of the internal standard areas, as well as the area ratios increased. The internal standard areas continued to decrease after the septum change. The internal standard area and the measured sample increased significantly after the exchange of both the septum and syringe and the standard deviations decreased significantly.

Typical results of the measurement sequence with the unsuitable septum are shown in figures 5 and 6. Each point is one measurement result. The use of the unsuitable septum meant the measurement process left PSS. The standard deviation of the peak area ratio, as well as the internal standard area, increased and systematic error became significant.

Comparing figure 5 with figure 6 it can be seen that the proposed method indicates the absence of the PSS in

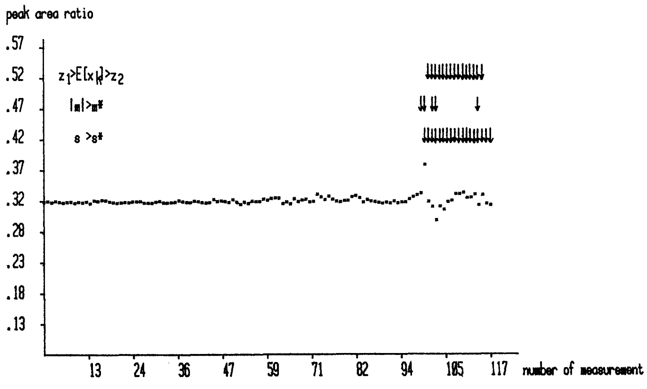

*Figure 5. The results of PSS test investigation to the peak area ratios (unsuitable septum for the task). The arrows correspond to failure of the steady state.*

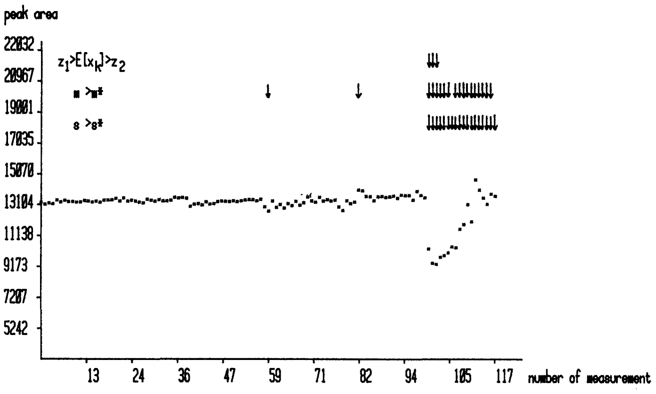

*Figure 6. The results of PSS test investigation to the peak area of internal standard (unsuitable septum for the task). The arrows correspond to failure of the steady state.*

nearly the same way for the internal standard area as it does for the peak area ratio.

### Conclusions

The measurement error model described here does not contain an autocorrelation-type measurement error component. The terms of the error model were divided into systematic error components and random error components, the sources of measurement error were disregarded. These components were separated on the basis of the mathematical characteristics of measurement error components. Currie has produced a more detailed model which takes into consideration the sources of analytical measurement error [9]; according to Currie the relationship between the unknown true value $\underline{x}$ to the experimental result $\hat{\underline{x}}$ determined in the course of analytical chemical measurement is

$$\hat{x} = x + \Delta + \delta + b + f(w)$$

where $\Delta$ is the constant portion of the systematic error; $\delta$ is the random error; $b$ is an erratic blunder or mistake; $f(w)$ is a 'lack of control term' which results from an external variable ($w$).

The final two kinds of measurement error must be eliminated with validation and statistical control of measurement results. This is called controlled measurement and in this case the systematic error and the random error have to be taken into account in a similar way to equation (1).

While on the basis of the investigation of analytical information (peak area ratios of control samples referred to internal standard) the measurement process state is evaluated without error, the measurement results of the internal standard characterize the measurement process with error.

The internal standard is added to the sample in order to increase the accuracy and reproducibility of the quantitative analysis. Therefore the analytical information does not include the effect of many potential sources of error (for example those arising from sample preparation) the measurement results of the internal standard, of course, are responsive to these effects. This correction is most useful in quantitative analyses. Using the measurement results from the internal standard is limited to the control of steady state with respect to the analytical information. If the internal standard is measurement results are used to discover the state of the measurement process, then two kinds of error have to be taken into account. A second order error from the statistical test may appear: the measurement process is assumed to be in non-PSS even if it can be considered to be in PSS on the basis of the analytical information (peak area ratios). Also a first order error from statistical test can appear—this would be an error in sample preparation.

The measurement results of the internal standard can indicate possible sources of the disturbance, for example a continued decrease of the internal standard peak area could mean that the syringe has failed and there is a sample loss during sampling. In this case the measurement process can be considered to be in PSS, with respect to the analytical information, but the signal-to-noise ratio decreases because of the decrease of the sample signal, resulting in decreasing sensitivity and detection limit.

Therefore, monitoring the PSS for the internal standard peak area allows the end of the measurement process steady state to be predicted. Control based on the measured results of the internal standard peak area is useful, but adequate for detecting measurement process PSS only with additional information—for example measuring control samples.

The method described was adequate for the separate determination of the simultaneously present systematic error and random error.

### References

1. Himmelblau, D. M., *Fault detection and Diagnosis in Chemical and Petrochemical Processes* (Elsevier, Amsterdam, Oxford and New York, 1978).
2. Pau, L. F., *Failure Diagnostic and Performance Monitoring* (Marcel Dekker Inc., New York and Basel, 1981).
3. Guillemin, C. L., *Journal of Chromatography*, **239** (1982).
4. Guillemin, C. L., *Journal of HRC and CC*, **3** (1980), 620.
5. Veress, G. E., Bezegh, A. and Pungor, E., In *Progress in Cybernetics and Systems Research* (Vol. 11., Hemisphere Publishing Corporation, Washington, D.C., 1982).
6. Gihman, I. I. and Shorohod, A. V., *Introduction to the Theory of Random Process* (Saunders, Philadelphia, 1969).
7. Almásy, G. A. and Hangos, K. M., *AMSE Athens Summer Conference on Modelling and Simulation* (Athens, 1984).
8. Eykhoff P. (Ed), *Trends and Progress in System Identification* (Pergamon Press, Oxford, 1981).
9. Currie, L. A., In *Treatise on Analytical Chemistry*, edited by I. M. Kolthoff and P. J. Elving (Part 1, Vol. 1, 2nd edn, John Wiley & Sons, New York, 1978), 95.

